# Knowledge, attitude and practice towards antibiotic use and resistance among the veterinarians in Bangladesh

**DOI:** 10.1371/journal.pone.0308324

**Published:** 2024-08-13

**Authors:** Md Samun Sarker, Sanjib Chandra Nath, Iftekhar Ahmed, Nure Alam Siddiky, Shariful Islam, Md Ehsanul Kabir, Zamila Bueaza Bupasha

**Affiliations:** 1 Bangladesh Livestock Research Institute (BLRI), Antimicrobial Resistance Action Center (ARAC), Savar, Dhaka, Bangladesh; 2 Department of Pharmacy, Jahangirnagar University, Dhaka, Bangladesh; 3 Institute of Epidemiology, Disease Control and Research (IEDCR), Mohakhali, Bangladesh; University of Lincoln - Brayford Campus: University of Lincoln, UNITED KINGDOM OF GREAT BRITAIN AND NORTHERN IRELAND

## Abstract

**Background:**

The emergence of antimicrobial resistance is a growing human and animal health concern around the world. When a number of studies have emphasized the Knowledge, Attitude and Practice (KAP) regarding antibiotic use and resistance in humans, little attention has been paid to the veterinary sector. The aim of this study was to understand the KAP towards antibiotic use and resistance among veterinarians in Bangladesh.

**Methods:**

A cross-sectional online based questionnaire survey was conducted from August to September 2020 among the registered veterinary practitioners. A self-administered Google form questionnaire consisting of 45 questions on knowledge, attitude and practice regarding antibiotic use and resistance was used. For statistical significance test we used Chi-square or Fisher’s Exact test and logistic regression for factor analysis.

**Results:**

A total of 208 registered veterinarians participated in this study. The study involved a majority of male participants, constituting 85.1%, while 54.8% of the participants held a Masters degree. Around 52% of the veterinarians were poultry practitioners. All respondents were familiar with antimicrobials. The participants (91.4%) knew that antibiotics cannot cure viral infections, while 97.6% believed that frequent antibiotic prescriptions rendered them less effective. Participants claimed that only they were eligible to prescribe drugs for the treatment of animals, and around 80% disagreed with adding antibiotics with feed/water as a growth promoter in livestock. Of the total participants, 87% believed that a local antimicrobial guideline would be more effective than an international one. A multivariable analysis revealed that male veterinarians have 2.37 times higher knowledge regarding antimicrobial use (AMU) and antimicrobial resistance (AMR) (OR = 2.37, CI = 1.01–5.59, *p* = 0.05). Veterinarians engaged in government services demonstrated a 2.59 times more favorable attitude towards AMU and AMR (OR = 2.59, CI = 0.99–6.73, *p* = 0.05). Additionally, respondents aged 31–35 were 0.45 times more likely to exhibit good practices (OR = 0.45, CI = 0.2–1.00, *p* = 0.05). However, gaps in practices were highlighted in our study, suggesting training deficiencies.

**Conclusion:**

The present study, for the first time conducted in Bangladesh, dictates that future interventions like academic courses, workshops, and seminars on antibiotic usage and resistance are needed to facilitate the knowledge, behavior and practice of veterinarians with regard to the rational use of antibiotics.

## Introduction

The global development of intensive farming has led to an upsurge in antimicrobial use (AMU) that led to the emergence and spread of antimicrobial resistance (AMR) [1]. Antibiotics are used as therapeutic as well as growth promotion purposes in animal farming practices in Bangladesh. One of the main factors influencing the development of AMR in animals is thought to be the overuse of antibiotics [2]. Antibiotic usage in animals is extremely prevalent globally and is predicted to increase by 67% by 2030 [1]. Most antibiotics are used in both human and animal interfaces, so the emergence of resistance through veterinary use is likely to have consequences on human health [3–5]. Bangladesh has been experiencing a high incidence of AMR [6,7]. Misuse and abuse of antibiotics are common both in humans and in animals in Bangladesh [6,8–10]. A study involving 73 poultry farms in Bangladesh reported the usage of antibiotics without prescriptions from registered veterinarians. Additionally, the investigation detected residual antibiotics in 26% of the tested samples, including breast, thigh, and liver [10]. Another study revealed that the majority of the antibiotics used in poultry farms fall under the Watch and Reserve group rather than Access [11]. Antibiotics in the Watch group have a greater resistance potential and comprise the majority of the top priority agents among the critically important antimicrobials for human medicine that are at high risk of bacterial resistance. Reserve group antibiotics should be reserved for the treatment of infections caused by multidrug-resistant organisms, whether confirmed or suspected. Antibiotics in the access group are active against a broad variety of regularly encountered susceptible infections. The findings of other studies identified that registered veterinarians also widely prescribed higher classes of antibiotics for treating healthy and unhealthy poultry flocks [12,13].

The WHO recommends an overall reduction of medically important antimicrobial use in food-producing animals, as well as a complete cessation of disease prevention and growth promotion [14]. The Government of Bangladesh enacted the “Animal and Fish Feed Act 2010”, which prohibited the use of all antibiotics in animal and fish feed in 2010 [15]. Still subsequent studies showed that such laws were not properly enforced, and the use of antibiotics with animal feeds is quite common [8–10]. These studies showed that unless raising awareness, motivation and ownership among veterinarians, farmers, feed sellers, and drug sellers, misuse of antibiotics in livestock will most likely continue to persist.

Any change in practice must start with the animal healthcare providers, followed by changes in antibiotic usage among the farmers. To formulate effective and sustainable strategies, recommendations, and treatment guidelines that optimize therapeutic efficacy and mitigate AMR in both human and animal health, it is imperative to assess the knowledge, attitude, and practices (KAP) of veterinary practitioners. Many countries have already conducted similar studies to go deep into this issue concerning both humans and animals [16–20]. For example, a KAP study conducted among dutch veterinarians revealed that good knowledge and positive attitude towards AMR positively impact the AMU and had positive influence among farmers [21]. Another KAP study conducted on veterinarians and para-veterinarians in Bhutan identified that most animal health workers had a poor knowledge on AMU and AMR leading to inappropriate use of antibiotics [22]. Similar findings were observed among university students on antibiotic usage in Nepalese University [23].

To the best of our knowledge, this study represents the first of its kind in investigating KAP on antibiotic use and resistance among Bangladeshi veterinarians. Although a study on the KAP of veterinary students in Bangladesh regarding antibiotic use and resistance has been previously reported [24]. Therefore, the study aimed to explore the KAP of the veterinarians of Bangladesh regarding antibiotic use and resistance.

## Methods

### Study design

A cross-sectional study was conducted from August to September 2020 using a self-administered questionnaire. Using purposive sampling, data collection was conducted among the registered veterinary practitioners of Bangladesh listed by the Bangladesh Veterinary Council (BVC), a government regulatory body of veterinary legislation and certification for veterinary practices in Bangladesh. To date, there are 7536 registered veterinarians in Bangladesh. BVC-listed veterinarians are the only certified professionals to treat and prescribe antibiotics for animals. Our target population was the registered veterinarians actively engaged in poultry, pet, and small and large animal practice throughout the country. Through social media (Facebook, LinkedIn and WhatsApp), the questionnaire was posted and circulated in different veterinary professional groups. The social media-based survey was launched on August 05, 2020. In mid-September 2020, the online questionnaire link was also messaged via mobile and Facebook messenger text and emailed to each registered veterinarian to boost the response rate. Regarding the sample size, the aim was to include as many veterinarians as possible.

### Questionnaire development and data collection

An online-based approximate 20–30 minutes long questionnaire was developed using the Google Forms platform by a multidisciplinary team consisting of microbiologists, public health specialists and epidemiologists. The questionnaire comprised four sections: the first one consisted of the demographic information of the veterinarians, the second section included 13 questions on knowledge of antibiotics and AMR, the third section contained 14 questions on attitudes, and the fourth section had 18 questions on practices regarding antibiotic use and their resistance. The majority of the answers were in multiple-choice format. The questionnaire was pretested among the veterinarians at the Antimicrobial Resistance Action Center (ARAC), Bangladesh Livestock Research Institute (BLRI), to validate and finalize the questionnaire. Finally, minor changes have been made in compliance with the participants’ responses and were circulated to the registered veterinarians. All the participants from ARAC in the pilot study were reminded not to participate in the final survey. Respondents were asked to read the consent paper made aware of the purpose of the study and requested to provide consent before participating in the survey.

### Ethical considerations

The study protocol was reviewed and approved by the ARAC, Animal Health Research Division, BLRI, Bangladesh (Approval no: 05/06/2020:06). Participation in the survey was completely non-compulsory and unpaid. If participants were willing to participate in the survey, they were first asked to click the “Yes” button as consent before continuing to the next sections. To prevent the loss of confidentiality, no identifying information of the participant was collected. This data was only accessible to the research team and investigators.

### Statistical analysis

Quantitative data were entered into MS Excel-2019 (Microsoft Corporation, Redmond, WA 98052, USA) for editing, sorting, and coding prior to statistical analysis. The statistical analysis was performed using STATA/IC 18 (StataCorp, College Station, TX, USA). To measure the internal consistency of the questionnaire, Cronbach’s alpha was employed. According to Taber [25], an acceptable range for Cronbach’s alpha coefficient is 0.45–0.98. In this study, the overall Cronbach’s alpha of the questions was 0.61, indicating acceptable internal consistency for each section of the questionnaire. Descriptive analysis was conducted to calculate the frequency and percentage of responses regarding KAP. or the KAP questions, a score of “1” was assigned for correct answers and “0” for incorrect answers. For each respondent, a total score was calculated. Respondents scoring above the mean (>12.99) were categorized as having “Correct” knowledge, while those scoring equal to or below the mean (≤12) were categorized as having “Incorrect” knowledge about antimicrobial resistance (AMR) and antimicrobial use (AMU). Similarly, for the attitude questions, scores above the mean (>12.95) were considered “Favorable,” and scores equal to or below the mean (≤12.95) were considered “Unfavorable” towards AMR and AMU. For practice questions, the mean score was 9.97, with scores above 9.97 categorized as “Good” practice and scores of 9.97 or below categorized as “Bad” practice.

For statistical significance test between the individual KAP questions and demographic factors (Age, Field of expertise, Type of service and Years of experience), chi-square tests were utilized. Fisher’s exact test was applied in instances where more than 20% of cells had expected frequencies less than 5. Subsequent univariate analyses assessed the associations between the binary outcome variables of knowledge (correct vs. incorrect), attitude (favorable vs. unfavorable), and practice (good vs. bad) concerning antimicrobial resistance (AMR) and antimicrobial use (AMU) and the independent variables, including gender, age, field of expertise, type of service, and years of experience. Finally, multivariable logistic regression analyses were conducted to determine the associations between the KAP outcomes and respondents’ demographics. The outcome variables for knowledge, attitude, and practice were coded as “1” for correct/favorable/good outcomes and “0” for incorrect/unfavorable/bad outcomes. The results were expressed as odds ratios (ORs) with 95% confidence intervals (95% CIs) and p-values.

## Results

### Participants’ characteristics

A total number of 208 veterinarians responded and took part in the questionnaire survey from eight administrative divisions of Bangladesh. Most of the participants were male (177/208; 85.1%), and 93.8% (195/208) were at the age from 25–35 years old. About 44% (92/208) had a Doctor of Veterinary Medicine (DVM) degree, while about 55% (114/208) had a Master’s degree. Half of the veterinarians were poultry practitioners, and the rest were pet animals, and large and small animal practitioners. Around 31% (65/208) had an experience of greater than 5 years. Detailed characteristics of the participants are presented in Table 1.

**Table 1:** Sociodemographic characteristics of the participants.

Characteristic	Number (n)	Percentage (%)
**Gender**		
Male	177	85.1
Female	31	14.9
**Education**		
DVM	92	44.2
Masters	114	54.8
PhD	2	0.96
**Age**		
25–30 Years	109	52.4
31–35 Years	86	41.4
Above 36 Years	13	6.3
**Field of expertise**		
Poultry practitioner	108	51.9
Pet animals’ practitioner	18	8.7
Large & Small Animals’ Practitioner	82	39.4
**Type of Service**		
Private	155	74.5
Governmental	53	25.5
**Years of practice**		
<1 Year	39	18.8
1–3 Year	63	30.3
>3–5 Years	41	19.7
Above 5 Years	65	31.3
**Job location (Division)**		
Barisal	8	3.9
Chittagong	71	34.1
Dhaka	59	28.4
Khulna	3	1.4
Mymensingh	15	7.2
Rajshahi	30	14.4
Rangpur	17	8.2
Sylhet	5	2.4

### Knowledge on antibiotics and AMR

The evaluation of veterinarians’ knowledge on antimicrobial resistance (AMR) and antimicrobial use (AMU) involved 14 questions. The results revealed that a significant majority (75%) of the veterinarians possessed correct knowledge. Although nearly all respondents were familiar with antimicrobials and antibiotics, 17.3% were unaware that antibiotics are different from antimicrobials. While 91.4% of the participants understood that antibiotics could not cure viral infections, 33.6% believed the use of antibiotics would speed up recovery from the common cold, cough, and other viral infections. The vets (47.6%) who had experienced between 1 to 3 years of practice significantly (p = 0.03) thought antibiotics would be effective against the common cold, cough and viral infections compared to the vets with other experienced groups. All of the vets were aware of antibiotic resistance, and 97.6% recognized that frequent prescriptions of antibiotics could render them less effective. However, some practitioners (6.7%) were unaware of the concept of antibiotic susceptibility testing. In addition, all vets, regardless of age, field of expertise, type of service and experience, believed biosecurity and improved hygiene could reduce the use of antibiotics (S1 Table). In the univariable analysis, a significant difference (*p* = 0.05) in knowledge levels was observed between male and female veterinarians (Table 2). The multivariable regression model indicated that male veterinarians had a significantly higher knowledge score compared to female veterinarians, with males being 2.37 times more knowledgeable (OR = 2.37, CI = 1.01–5.59, p = 0.05) (Table 3).

**Table 2:** Test of statistical significance of the variations in the respondents’ knowledge, attitudes, and practices by their characteristics (N = 208).

Variables	Categories	Knowledge			Attitude			Practice		
		Correct n (%)	Incorrect n (%)	*p*-value	Favorable n (%)	Unfavorable n (%)	*p*-value	Good n (%)	Bad n (%)	*p*-value
Gender	Male	137 (77.4)	40 (22.6)	0.05	125 (70.6)	52 (29.4)	0.13	108 (61)	69 (39)	0.72
	Female	19 (61.3)	21 (38.7)		26 (83.9)	5 (16.1)		20 (64.5)	11 (35.5)	
Age	25–30	83 (76.2)	26 (23.8)	0.85	80 (73.4)	29 (26.6)	0.65	74 (67.9)	35 (32.1)	0.14
	31–35	64 (74.4)	22 (25.6)		63 (73.3)	23 (26.7)		47 (54.7)	39 (45.3)	
	>36	9 (69.2)	4 (30.8)		8 (61.5)	5 (38.5)		7 (53.9)	6 (46.1)	
Field of expertise	Poultry	79 (73.2)	29 (26.8)	0.71	67 (62)	41 (38)	<0.001	70 (64.8)	38 (35.2)	0.42
	Pet	13 (72.2)	5 (27.8)		18 (100)	0 (0)		12 (66.7)	6 (33.3)	
	Large & small animals	64 (78.1)	18 (21.9)		66 (80.5)	16 (19.5)		46 (56.1)	36 (43.9)	
Type of service	Private	116 (74.8)	39 (25.2)	0.93	105 (67.7)	50 (32.3)	0.007	96 (61.9)	59 (38.1)	0.84
	Government	40 (75.5)	13 (24.5)		46 (86.8)	7 (13.2)		32 (60.4)	21 (39.6)	
Years of experience	<1–3	76 (74.5)	26 (25.5)	0.37	76 (74.5)	26 (25.5)	0.07	65 (63.7)	37 (36.3)	0.79
	>3–5	34 (82.9)	7 (17.1)		34 (82.9)	7 (17.1)		25 (61)	16 (39)	
	>5	46 (70.8)	19 (29.2)		41 (63.1)	24 (36.9)		38 (58.5)	27 (41.5)	

**Table 3:** Logistic regression analysis of the demographic factors associated with veterinarians’ knowledge, attitudes, and practices on AMU and AMR.

Variables	Categories	Knowledge	Attitude	Practice
		OR, 95% CI, *p*	OR, 95% CI, *p*	OR, 95% CI, *p*
Gender	Female	Ref	Ref	Ref
	Male	2.37, 1.01–5.59, 0.05	0.76, 0.25–2.28, 0.62	0.78, 0.34–1.82, 0.57
Age	25–30	Ref	Ref	Ref
	31–35	0.89, 0.36–2.24, 0.81	1.64, 0.63–4.27, 0.31	0.45, 0.2–1.00, 0.05
	>36	0.84, 0.17–4.19, 0.83	1.2, 0.24–5.86, 0.83	0.40, 0.09–1.7, 0.21
Field of expertise	Poultry	Ref	Ref	Ref
	Pet	1.21, 0.36–4.11, 0.76	1.13, 0.67–2.76, 0.32	0.88, 0.28–2.75, 0.83
	Large & small animals	1.55, 0.71–3.39, 0.28	1.76, 0.82–3.76, 0.15	0.64, 0.32–1.28, 0.21
Type of service	Private	Ref	Ref	Ref
	Government	0.9, 0.38–2.07, 0.8	2.59, 0.99–6.73, 0.05	1.18, 0.56–2.48, 0.67
Years of experience	<1–3	Ref	Ref	Ref
	>3–5	1.8, 0.65–4.9, 0.26	1.66, 0.59–4.63, 0.34	1.12, 0.52–2.85, 0.66
	>5	0.9, 0.33–2.72, 0.91	0.54, 0.19–1.50, 0.26	1.48, 0.57–3.85, 0.43

### Attitude towards antibiotic use and resistance

**Fig 1:**
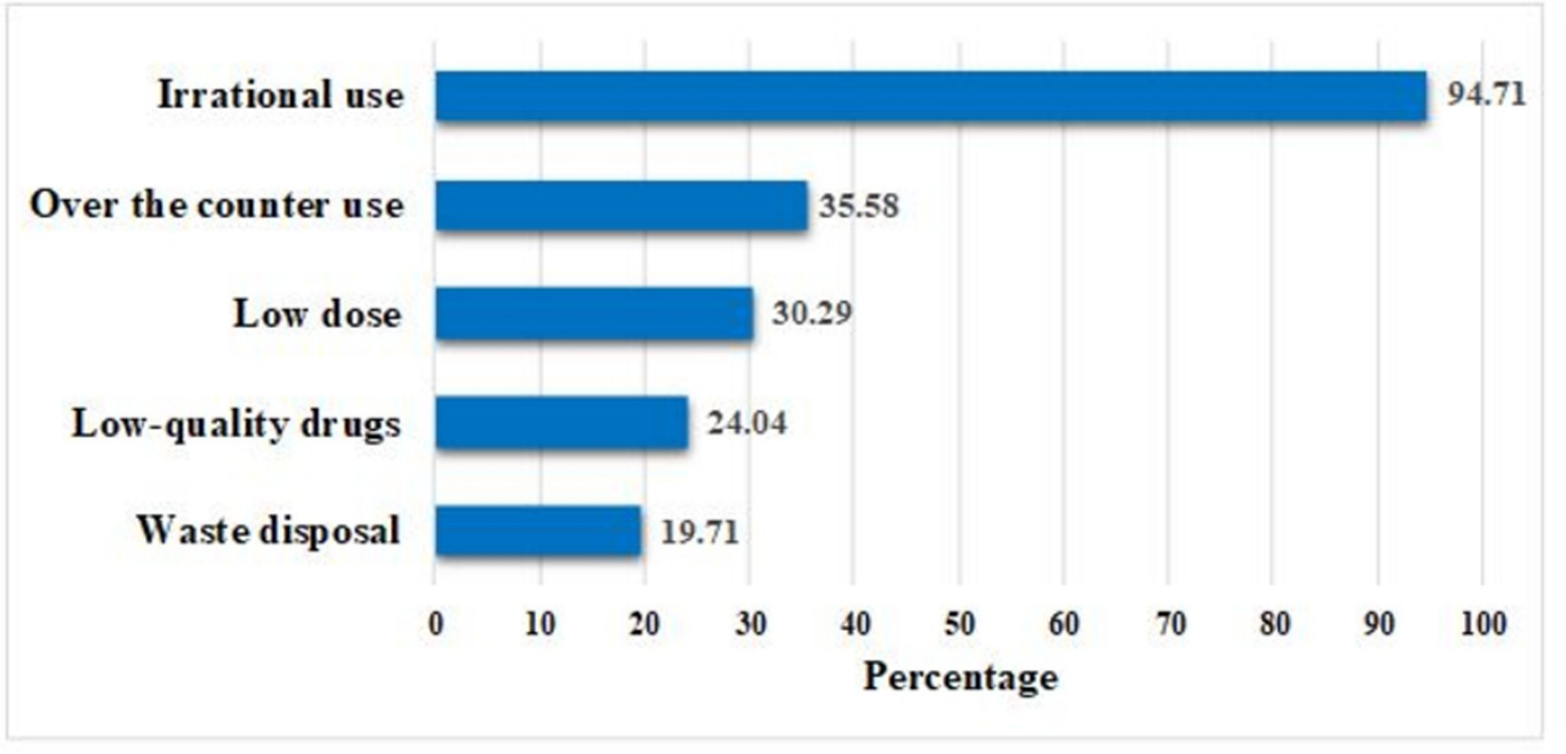
Major reasons of AMR indicated by the veterinarians.

A significant proportion (72.6%) of veterinarians demonstrated a favorable attitude towards AMR and AMU. Out of the 208 participants, 207 opinioned that only veterinarians are eligible to prescribe drugs for animals. Moreover, nearly all agreed that antibiotic abuse is prevalent in veterinary practices in Bangladesh. Practitioners also had a positive attitude towards vaccination for the purpose of preventing diseases and reducing the use of antibiotics in animals. Most practitioners (99%) felt that a national guideline on rational antibiotic use is necessary, and 87% believed a local antimicrobial guideline would be more useful than an international one. Around 80% disagreed with adding antibiotics with feed/water as a growth promoter in poultry and livestock. Regarding the major reasons for antibiotic resistance, irrational use of antibiotics was identified as the primary cause by 94.71%, followed by over-the-counter use, low dose, low-quality antibiotics, and waste disposal of antibiotics (Fig 1). Around 98% of vets agreed that an inappropriate or half course of antibiotics leads to antibiotic resistance. A significant difference between different age groups (p <0.001) was observed for the attitude "inappropriate use or half course of antibiotics leads to antibiotic resistance". Significant attitudes were also observed among different categories of “Years of practice” concerning the severity of a disease if an individual (p = 0.02) or animal (p = 0.03) could not be treated with antibiotics. The lack of training or workshop or seminar regarding antimicrobial resistance was seen in the middle-aged vets (43%). In addition, attending training or workshops or seminars on antimicrobial resistance varied significantly across different groups of fields of expertise (p = 0.01), type of service (p = 0.01), and years of practice (p = 0.01) (S2 Table). Factor analysis for the attitude score revealed significant differences among different types of services (*p* = 0.007) and fields of expertise (*p* < 0.001) (Table 2). It was observed that government veterinarians have 2.59 times more favorable attitudes compared to veterinarians working in the private sector (OR = 2.59, CI = 0.99–6.73, *p* = 0.05) (Table 3).

### The practice of the veterinarians regarding antibiotic prescribing

**Fig 2:**
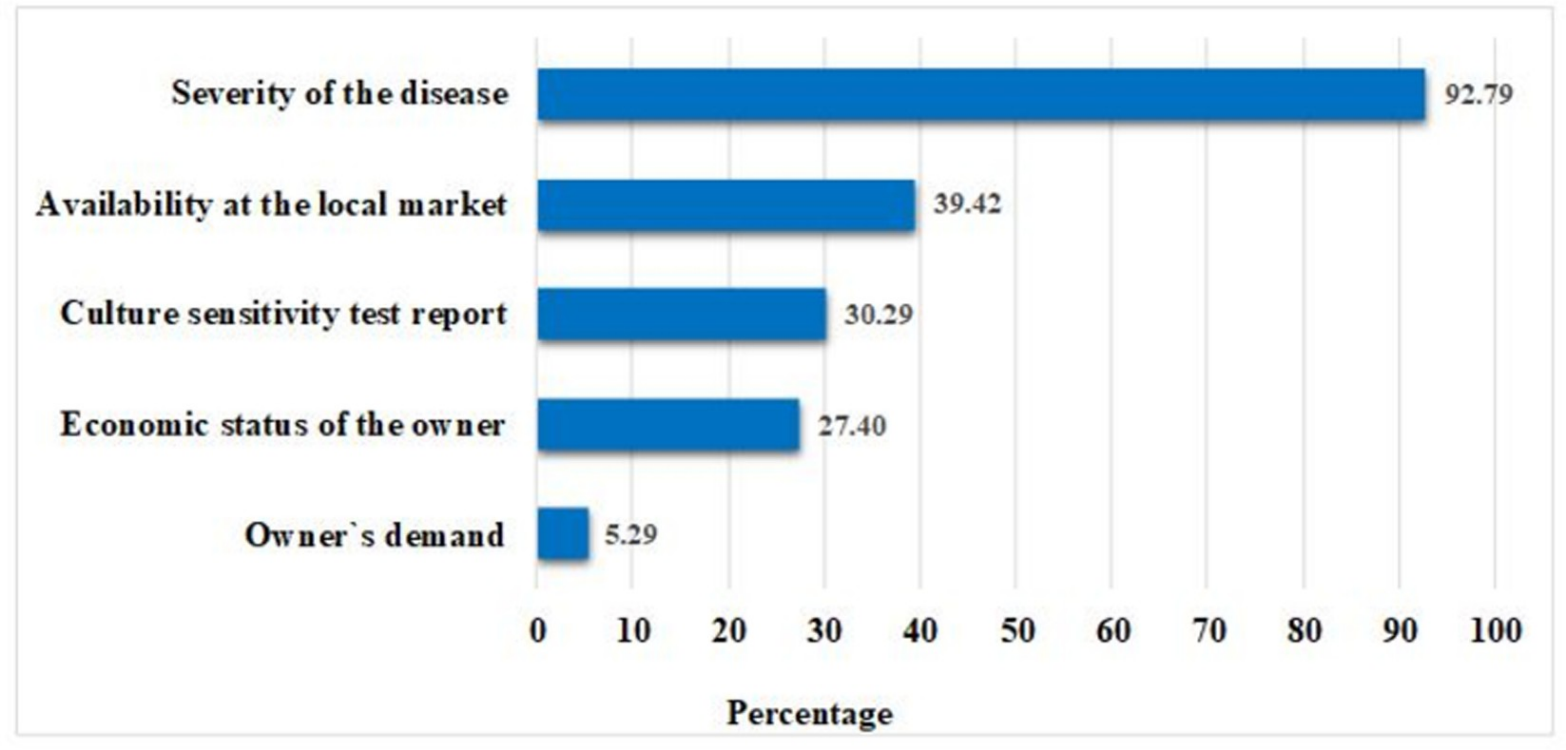
Factors considered by veterinarians while prescribing antibiotic.

A total of 18 practice related questions were used in the survey, with the distribution of responses from participants is presented in S3 Table. The majority (70.2%) of the veterinarians reported that they sometimes prescribe antibiotics over the phone or without examining the animals. Also, only 9.1% of the practitioners always or often recommend antimicrobial susceptibility testing before prescribing antibiotic agents. Half of the participants prefer broad-spectrum antibiotics, while the other half prefer narrow-spectrum antibiotics. Results also showed that antibiotics constitute a large percentage of daily prescribed drugs. Moreover, combined antibiotic therapy is also preferred to single therapy by about 65% of the practitioners. Old-generation antibiotics are preferred to new-generation antibiotics by most (63.5%) as a first-line treatment. Some practitioners (26%) reported prescribing antibiotics without determining the body weight of the animals. Most practitioners (74.5) do not mention the antibiotic withdrawal period in their prescriptions. When exploring the factors considered by the vets while prescribing antibiotics, the severity of the disease was found to be the most important factor. The vets also considered other factors such as the availability of an antibiotic in the local market, culture sensitivity test report, economic status of the owner, and owners’ demand for antibiotics (Fig 2). There was significant variation in practitioners’ age for the practice- prescribing antibiotics in self-limiting infection (p = 0.03) and suggesting vaccination for disease prevention (p = 0.01). For the field of expertise, a significant difference was observed for the practices- suggesting antimicrobial susceptibility testing (p = 0.03), poor clinical response due to AMR (p = 0.04), mention withdrawal period (p = 0.03), and combined antibiotic therapy (p = 0.05). In the case of type of service- antimicrobial sensitivity testing facility (p = 0.03), percentage of antibiotics in prescriptions (p = 0.05), and drug’s mechanism of action (p = 0.03) have a significant difference. On the other hand, significant variation in years of experience was found for the practice- antimicrobial sensitivity testing facility (p = 0.03) (S3 Table). Finally, in terms of demographic factors associated with good practice regarding AMR and AMU, we found that veterinarians who were 31–35 years old perform 0.45-time good practice compared to the age group 25–30 years old (OR = 0.45, CI = 0.2–1.00, *p* = 0.05). Further, veterinarians experienced in large and small animal practice were found to have 0.64-time good practice score compared to poultry practitioners (OR = 0.64, CI = 0.32–1.28, *p* = 0.21) (Table 3).

## Discussion

The present study investigates the knowledge, attitude, and practice of Bangladeshi veterinary practitioners regarding antibiotic use and antimicrobial resistance. The present study investigated the knowledge, attitudes, and practices of Bangladeshi veterinary practitioners regarding antibiotic use and antimicrobial resistance. The reduction of AMR in the animal industries sector requires intervention from all stakeholders (e.g., veterinary students, para-vets, drug and feed sellers, and farmers), and especially from veterinarians [26].

The demographic information revealed that most participants were male (85.1%), with a smaller proportion of female participants (14.9%). This gender distribution aligns with studies conducted among veterinarians in Bangladesh [26] and Nepal [27], which reported male participants at 83.3% and 85%, respectively. The lower number of female veterinarians engaged in clinical practice, observed in both Bangladesh and Nepal, may be attributed to the tendency of female veterinarians to pursue roles in administration and academia rather than clinical practice [27]. Furthermore, the majority of the veterinarians were aged 25–30 years (52.4%), and only 25.5% of the participants were employed in government service. This age distribution is similar to findings from Nepal, where 55.4% of veterinarians fell within the 24–30 age group, and a relatively low percentage were from government service (29.1%). However, a study in Bangladesh found that more than half of the veterinarians (53.9%) were employed in government service [26], indicating a higher prevalence of government employment among veterinarians in that particular study.

It was found that veterinarians have good knowledge regarding AMR and AMU. However, some vets had gaps in knowledge of certain crucial concepts; for example, 17.3% of the practitioners’ considered antibiotics and antimicrobials to be the same. Failure to differentiate between antimicrobials, and antibiotics and their roles can be a major reason for inappropriate antibiotic prescribing such as prescribing antibiotics for viral infections. Another surprising finding was that although most knew that antibiotics could not cure viral infections, one-third of the vets believed antibiotics would speed up colds, coughs, or other viral infections. Similar finding was observed in a study in Nepal where a quarter of the participants believe that antibiotics can be used in all type of infections [28]. However, there is no evidence that antibiotics can cure viral infections or speed up the recovery of viral infections such as the common cold [29,30]. We found about 61.1% of the respondents attended training or seminar or workshop on AMR, which was higher than a previous study (47.2%) in Nigeria [31].

Our study revealed that majority (72.6%) of the veterinarians showed favorable attitude towards AMR and AMU. Specially, 79.3% mentioned that they did not use antimicrobials as growth promoters or feed additives, and 98.6% suggested vaccination against preventable disease which was similar to the findings of Napal (86.5% and 89%) [27]. Nearly all veterinarians (98.6%) were aware of the antibiotic withdrawal period and considered it important to maintain an appropriate withdrawal period prior to selling animals treated with antibiotics in order to avoid antibiotic residues in animals, and this finding is in line with the study conducted in Nigeria [32]. However paradoxically, while prescribing antibiotics, only one out of four practitioners mentioned the withdrawal period in the prescriptions. This may happen because practitioners do not have the knowledge of the withdrawal periods of the specific antibiotics they prescribe or because they think the farmers will not understand or follow instructions related to withdrawal periods anyway. Besides, previous studies have shown that most Bangladeshi farmers do not have knowledge of the antibiotic withdrawal period [10,11]. Hence, non-adherence to the required withdrawal periods by either party may result in the presence of residual antibiotics in food and animal products [33]. Antibiotic residues can be toxic to humans as well as may contribute to the development of AMR [33,34].

Prescribing antibiotics based on the results of susceptibility testing is recommended to make sure that the prescribed regimen is effective against the infection. It is concerning that such practice was not often followed by a good number (40.9%) of practitioners even after the initial treatment failed which is in line with the study conducted in Bangladesh [26]. However, these findings were contradicting the observations of other studies [35–37], where majority of the veterinarians practiced antibiotic susceptibility test (AST) in the selection of antimicrobials. This lower number of performing AST can partially be explained by the fact that most areas of Bangladesh did not have any facility to test antimicrobial sensitivity, as reported by the veterinarians, and higher cost associated with existing AST facilities [38]. The absence of susceptibility data can also promote inappropriate use of antimicrobials in terms of using combined antibiotic therapy since the vets may want to prescribe more than one drug to maximize the chance of therapeutic success with the hope that if one drug is found ineffective, the others will work [24,39]. A study of veterinary surgeons in the United Kingdom also reported similar findings where the surgeons only occasionally carried out susceptibility testing [40]. Another concerning practice is to prescribe antibiotics over phone without clinical examination of the animals also noted in another study in Bangladesh [26], and this is considered as a bad practice more pressing the AMR situation in Bangladesh [22,36].

The use of antibiotics for disease prevention of animals by farmers and poultry dealers has been reported in Bangladesh [8], although such practices are not recommended [14]. A minor percentage (8.7%) of the veterinarians think that antibiotics should be used for disease prevention. On the other hand, study conducted in India reported higher percentage (39%) of veterinarians used antibiotics as prophylaxis, mainly for disease prevention. In case of choosing antimicrobials, half of the veterinarians prefer to prescribe narrow-spectrum antimicrobials than broad spectrum, which is much lower with studies conducted in Bhutan and Nepal [22,27]. Most veterinarians in this study do not consider the use of antimicrobials for disease prevention. Instead, the participants have shown a very positive attitude towards vaccination for both infection prevention and lowering the use of antibiotics. Given the fact that about half of the most significant animal diseases are of viral origins [41], vaccination can be very effective and efficient in lowering the occurrences of infectious diseases in animals and will subsequently confer financial gains to the farmers as well as help to minimize unnecessary use of antibiotics. Vaccines have also been recommended for infection prevention by WHO [14].

Participants were knowledgeable about antibiotic resistance, its causes, and its consequences. However, unless such knowledge is translated into practice, no real benefit will be achieved. We have identified a number of inappropriate practices by the veterinarians, including excessive antibiotic prescribing, prescribing antibiotics over the phone without examining animals, not relying on susceptibility testing, not mentioning the antibiotic withdrawal period in prescription, etc.

This survey revealed the varied differences in knowledge, attitude and practice of antibiotic use among different age groups of veterinarians in Bangladesh. It was not conclusively established the variation of predefined questions answered by the different age groups of vets. This study revealed that old-aged vet groups (31–35 years and >36 years old) performed 0.45 and 0.40-times good practice compared to the young age group (25–30 years old) [26,27]. This may be due to their work experiences over time and participation in trainings and seminars. However, studies conducted among veterinarians in India found that age group of <30 years and experience with <10 years have higher knowledge and attitude score compared to other groups [42]. Dutch veterinarians with more years of experience were found to be less concerned about the possible contribution of veterinary antibiotic use to antimicrobial resistance which is not in line with our study findings [43]. These findings indicate the necessity of interventions to enhance the awareness among young veterinarians, addressing the major aspects of AMR and AMU. To understand the perceptions and barriers, further investigation is required to appropriate the use of antibiotics in livestock and poultry among the vet’s subpopulation. It would help the policy makers and academicians to ensure proper training and impart practical field-based knowledge of appropriate use of antibiotics and AMR to the vet students, young vets and all level-aged groups of vets.

Another major problem is that Bangladeshi farmers rely more upon village doctors, traditional healers and drug sellers and consider government veterinarians as a last resort for seeking health services for their livestock [9]. This trend needs to change, and qualified veterinarians should be the primary source of advice in order to promote rational antibiotic use. Veterinarians should also play an active role in dispelling misconceptions of the farmers surrounding antibiotics, and they should adopt the appropriate practices. The government should focus on implementing the laws pertaining to the judicious use of antibiotics, as well as recruiting more qualified veterinarians so that farmers can have easy access to them.

## Limitations

A few limitations were witnessed during the conduct of the current study. The number of participants in the survey was low, which may be due to several factors such as unwillingness to participate or lack of internet access or long questionnaire or some questions seemed too difficult. Sometimes, respondents may have declined to share information they considered inappropriate or mistaken, resulting in an under-reporting of certain aspects of antibiotics and AMR knowledge and practices. The study could not meet the exact proportional number of respondents with anticipated geographic locations due to the freedom of choice of the respondents.

## Conclusions

The study revealed that majority of the veterinarians possesses a solid understanding of AMU and AMR. However, some participants lack clarity in distinguishing between antibiotics and antimicrobials, and also believe that antibiotics can hasten recovery of cold, cough and other diseases caused by common flu virus, leading to antibiotics misuse in animals. The study further identified concerning practices among veterinarians such as not mentioning withdrawal period in prescription, prescribing excessive antibiotic, prescribing antibiotics over the phone without examining the animals and without antibiotic susceptibility testing are aggravating the situation of AMR in the Bangladesh. The study findings suggest policy guidelines and advocacy to the public and private veterinarians in improving the prudent use of antibiotics. Antimicrobial stewardship programs in public and private veterinary hospitals need to be initiated to promote the rational use of antibiotics. Improved knowledge and awareness of veterinarians through continuous education and training can enhance the rational use of antibiotics. Ensuring the dissemination of regularly updated national antibiotic usages guidelines for food animals, along with fostering understanding of the pivotal role played by good biosecurity and vaccination practices in disease prevention, is paramount. Additionally, facilitating affordable antimicrobial susceptibility testing with easy accessibility is essential to effectively combat the escalating threat of antimicrobial resistance in the veterinary sector of Bangladesh.

## Supporting information

S1 Text Questionnaire for KAP survey on antibiotics and AMR. (DOCX)

S1 Table Veterinarian’s knowledge on antibiotic use and resistance. (DOCX)

S2 Table Veterinarian’s attitude towards antibiotic use and resistance. (DOCX)

S3 Table Veterinarian’s practice regarding antibiotic and resistance. (DOCX)

S1 Dataset (XLSX)
