# Supplementary material for: Knowledge, attitude and practice towards antibiotic use and resistance among the veterinarians in Bangladesh
Source: PLoS One. 2024 Aug 13;19(8):e0308324. doi: 10.1371/journal.pone.0308324 (PMC11321546; doi:10.1371/journal.pone.0308324)
Supplement: S1 Text — (DOCX) [file pone.0308324.s001.docx]

**S1 Text. Questionnaire for KAP survey on antibiotics and AMR.**

# A survey on Knowledge, attitude and practice towards antibiotic use and resistance among the veterinarians in Bangladesh

**Consent Paper**

We, a number of researchers from Bangladesh Livestock Research Institute (BLRI) have decided to run a survey entitled of “Knowledge, Attitude and Practice towards antibiotic use and resistance among the veterinarians in Bangladesh” to bring out the scenery of practitioner’s knowledge, attitude and practice regarding antibiotic use. Your participation will be accounted as a volunteer. If you are not interested to take part in this survey, then no need to be worried.

# Why this survey will be conducted?

This study aims to evaluate the knowledge, attitude and practice (KAP) of veterinary practitioners towards the antibiotic use in the Bangladesh.

# What will be your activities?

You will be answered a number of questions whatever you know. These answers will never be used to identify you.

# Will the provided data be confidential? Yes

If you have any query, please don’t hesitate to contact to Dr. Md Samun Sarker ([samuncvasu@gmail.com](mailto:samuncvasu@gmail.com)).

I read this consent paper thoroughly and take part in this survey willingly and also accept that the authority can publish this work without my concern.

As a consent please click the “Yes” button Yes No

# Socio-demographic characteristics of veterinary practitioners

1. E-mail address
2. Gender

- Male
- Female

1. Educational status

- DVM
- MS
- PhD

1. Age

- 25-30
- 31-35
- Above 36

1. Field of expertise

- Poultry practitioner
- Pet animals’ practitioner
- Small and large animals’ practitioner

1. Years of practice

- <1 Year
- 1-3 Year
- >3-5 Years
- Above 5 Years

1. Job Location (District)

# Knowledge of antibiotic use and resistance

1. Are you familiar with antimicrobials?
2. Yes
3. No
4. Are you familiar with antibiotics?
5. Yes
6. No
7. Do you think that antibiotic is different from antimicrobials?
8. Yes
9. No
10. Do you know about antibiotic withdrawal period?
11. Yes
12. No
13. Do you know about antibiotic susceptibility testing?
14. Yes
15. No
16. Do you know about antibiotic resistance?
17. Yes
18. No
19. Do you know any antibiotics that are prohibited to use in livestock?
20. Yes
21. No
22. Can antibiotics be used to cure infections caused by bacteria?
23. Yes
24. No
25. Can antibiotics be used to cure infections caused by virus?
26. Yes
27. No
28. Do you think the use of antibiotics will speed up recovery of cold, cough and other diseases caused by common flu virus?
29. Yes
30. No
31. Do you think frequent prescribe of antibiotics will decrease the efficacy of drug?
32. Yes
33. No
34. Do you think antibiotic should be used for disease prevention?
35. Yes
36. No
37. Do you think biosecurity and improved hygiene can reduce the use of antibiotics?
38. Yes
39. No

# Attitude of antibiotic use and resistance

- 1. In your opinion, only veterinarians are eligible for drugs prescriptions of animals?
     1. Yes
     2. No
  2. At present, there is abuse of antibiotics
     1. Yes
     2. No
  3. Antibiotic resistance affects you and your family’s health
     1. Yes
     2. No
  4. When a disease in individual can't be treated with antibiotics, how serious do you think it could be?
     1. Very serious
     2. Serious
     3. Less serious
     4. Not serious at all
  5. When a disease in animal cannot be treated with antibiotics, how serious do you think it could be?
     1. Very serious
     2. Serious
     3. Less serious
     4. Not serious at all
  6. Do you think vaccination can prevent disease?
     1. Yes
     2. No
  7. Do you think vaccination can reduce the uses of antibiotics?
     1. Yes
     2. No
  8. Is it necessary to establish a law on “Rational use of antibiotics” at the national level?
     1. Yes
     2. No
  9. A local antimicrobial guideline would be more useful than international one
     1. Yes
     2. No
  10. It is important to add antibiotic with feed/water as growth promoter in livestock
      1. Agree
      2. Neutral
      3. Disagree
  11. Inappropriate use or half course of antibiotics leads to antibiotic resistance
      1. Agree
      2. Neutral
      3. Disagree
  12. An appropriate withdrawal period is needed before selling to avoid antibiotic residue in food animal?
      1. Agree
      2. Neutral
      3. Disagree
  13. In your opinion, what are the major reasons of antibiotic resistance in Bangladesh?
      1. Irrational use
      2. Low dose
      3. Over the counter
      4. Waste disposal
      5. Under quality drugs
  14. Have you ever attended any training/conference/seminar/workshop on antimicrobial resistance?
      1. Yes
      2. No

# Practice of antibiotic use and resistance

1. Do you prescribe antibiotics over phone or without seeing/examining animals?
   1. Yes
   2. No
2. Do you have facilities in your area to test antimicrobial sensitivity?
   1. Yes
   2. No
3. How often on a first visit to a farm you carry out antimicrobial susceptibility testing if you suspect a bacterial infection is present?
   1. Often/Always
   2. Sometimes
   3. Never/rarely
4. How often do you carry out antimicrobial susceptibility testing, when a pathogen has not responded to the first antibiotic used?
   1. Often/Always
   2. Sometimes
   3. Never/rarely
5. How often do you encounter a poor clinical response to an antimicrobial used?
   1. Often/Always
   2. Sometimes
   3. Never/rarely
6. How commonly do you feel that poor clinical response may be due to antimicrobial resistance?
   1. Often/Always
   2. Sometimes
   3. Never/rarely
7. How often have you had to change an antimicrobial agent because of resistance confirmed on antimicrobial susceptibility testing?
   1. Often/Always
   2. Sometimes
   3. Never/rarely
8. What are the factors you consider for prescribing antibiotics?
   1. Owner`s demand
   2. Economic status of the owner
   3. Severity of the disease
   4. Availability at the local market
   5. Culture sensitivity test report
9. Which spectrum of antibiotics do you prefer most?
   1. Narrow spectrum
   2. Broad spectrum
10. What percentage of your daily prescriptions has antibiotics?

I. 20% to 40%

1. 40% to 60%
2. Above 60%
3. Do you mention withdrawal period in the prescription?
   1. Yes
   2. No
4. Do you suggest keeping drug register of animals?
   1. Yes
   2. No
5. Do you prefer combine antibiotics to ensure therapeutic success?
   1. Yes
   2. No
6. Do you administer antibiotics to animals without determining their body weight properly?
   1. Yes
   2. No
7. Do you consider whether an infection is self-limiting before prescribing antibiotics?
   1. Yes
   2. No
8. As the first line of treatment, do you choose new generation antibiotics rather than older generations like penicillin?
   1. Yes
   2. No
9. Do you consult with other veterinarian/other educational resources when in doubt of a drug’s mechanism of action?
   1. Yes
   2. No
10. Do you suggest clients to vaccinate their animals against preventable diseases?
    1. Yes
    2. No
