# Supplementary material for: Knowledge, attitude and practice towards antibiotic use and resistance among the veterinarians in Bangladesh
Source: PLoS One. 2024 Aug 13;19(8):e0308324. doi: 10.1371/journal.pone.0308324 (PMC11321546; doi:10.1371/journal.pone.0308324)
Supplement: S1 Table — (DOCX) [file pone.0308324.s002.docx]

**Table S2. Veterinarian’s knowledge on antibiotic use and resistance**

| **Knowledge statements** | **Overall**  **n (%)** | **Age groups (Year)**  **n (%)** | | | | **Field of expertise**  **n (%)** | | | | **Type of Service**  **n (%)** | | | **Years of practice**  **n (%)** | | | | |
| --- | --- | --- | --- | --- | --- | --- | --- | --- | --- | --- | --- | --- | --- | --- | --- | --- | --- |
|  |  | **25-30** | **31-35** | **>36** | **P value** | **Poultry** | **Pet animals** | **Large & small  animals** | **P value** | **Private** | **Government** | **P value** | **<1** | **1-3** | **>3-5** | **>5** | **P value** |
| K1. Do you know ‘antimicrobial’ (Ans-Yes) | | | | | | | | | | | | | | | | | |
| Yes | 208 (100) | 109 (100) | 86 (100) | 13 (100) |  | 108 (100) | 18 (100) | 82 (100) |  | 155 (100) | 53 (100) |  | 39 (100) | 63 (100) | 41 (100) | 65 (100) |  |
| No | 0 | 0 | 0 | 0 |  | 0 | 0 | 0 |  | 0 | 0 |  | 0 | 0 | 0 | 0 |  |
| K2. Are you familiar with the concept of antibiotics? (Ans-Yes)^a^ | | | | | | | | | | | | | | | | | |
| Yes | 207 (99.5) | 109 (100) | 85 (98.8) | 13 (100) | 0.48 | 107 (99.1) | 18 (100) | 82 (100) | 0.99 | 154 (99.4) | 53 (100) | 1.00 | 39 (100) | 63 (100) | 40 (97.6) | 65 (100) | 0.39 |
| No | 1 (0.5) | 0 | 1 (1.2) | 0 |  | 1 (0.93) | 0 | 0 |  | 1 (0.6) | 0 |  | 0 | 0 | 1 (2.4) | 0 |  |
| K3. Do you think that antibiotic is different from antimicrobials? (Ans-Yes)^a^ | | | | | | | | | | | | | | | | | |
| Yes | 169 (81.3) | 88 (80.7) | 70 (81.4) | 11 (84.6) | 0.91 | 89 (82.4) | 13 (72.2) | 67 (81.7) | 0.22 | 128 (82.6) | 41 (77.4) | 0.55 | 31 (79.5) | 52 (82.5) | 33 (80.5) | 53 (81.5) | 0.99 |
| No | 36 (17.3) | 20 (18.4) | 14 (16.3) | 2 (15.4) |  | 19 (17.6) | 4 (22.2) | 13 (15.9) |  | 25 (16.1) | 11 (20.8) |  | 7 (18) | 10 (15.9) | 8 (19.5) | 11 (17) |  |
| Blank/No Answer | 3 (1.4) | 1 (0.9) | 2 (2.3) | 0 |  | 0 | 1 (5.6) | 2 (2.4) |  | 2 (1.3) | 1 (1.9) |  | 1 (2.6) | 1 (1.6) | 0 | 1 (1.5) |  |
| K4. Do you know about antibiotic withdrawal period? (Ans-Yes)^a^ | | | | | | | | | | | | | | | | | |
| Yes | 206 (99.04) | 108 (99.1) | 85 (98.8) | 13 (100) | 0.97 | 106 (98.1) | 18 (100) | 82 (100) | 0.59 | 153 (98.7) | 53 (100) | 1.00 | 38 (97.4) | 62 (98.4) | 41 (100) | 65 (100) | 0.45 |
| No | 2 (0.96) | 1 (0.9) | 1 (1.2) | 0 |  | 2 (1.9) | 0 | 0 |  | 2 (1.3) | 0 |  | 1 (2.6) | 1 (1.6) | 0 | 0 |  |
| K5. Do you know about antibiotic susceptibility testing? (Ans-Yes)^a^ | | | | | | | | | | | | | | | | | |
| Yes | 193 (92.8) | 101 (92.7) | 82 (95.4) | 10 (76.9) | 0.05 | 99 (91.7) | 17 (94.4) | 77 (93.9) | 0.96 | 144 (92.9) | 49 (92.5) | 0.82 | 37 (94.9) | 59 (93.7) | 39 (95.1) | 58 (89.2) | 0.91 |
| No | 14 (6.7) | 8 (7.3) | 3 (3.5) | 3 (6.7) |  | 8 (7.4) | 1 (5.6) | 5 (6.1) |  | 10 (6.5) | 4 (7.6) |  | 2 (5.1) | 4 (6.4) | 2 (4.9) | 6 (9.3) |  |
| Blank | 1 (0.5) | 0 | 1 (1.2) | 0 |  | 1 (0.9) | 0 | 0 |  | 1 (0.7) | 0 |  | 0 | 0 | 0 | 1 (1.5) |  |
| K6. Do you know about antibiotic resistance? (Ans-Yes) | | | | | | | | | | | | | | | | | |
| Yes | 208 (100) | 109 (100) | 86 (100) | 13 (100) |  | 108 (100) | 18 (100) | 82 (100) |  | 155 (100) | 53 (100) |  | 39 (100) | 63 (100) | 41 (100) | 65 (100) |  |
| No | 0 | 0 | 0 | 0 |  | 0 | 0 | 0 |  | 0 | 0 |  | 0 | 0 | 0 | 0 |  |
| K7. Do you know any antibiotics that are prohibited to use in livestock? (Ans-Yes)^a^ | | | | | | | | | | | | | | | | | |
| Yes | 197 (94.7) | 105 (96.3) | 80 (93) | 12 (92.3) | 0.40 | 101 (93.5) | 17 (94.4) | 79 (96.3) | 0.37 | 146 (94.2) | 51 (96.2) | 0.21 | 38 (97.4) | 58 (92.1) | 40 (97.6) | 61 (93.9) | 0.73 |
| No | 10 (4.8) | 4 (3.7) | 5 (5.8) | 1 (7.7) |  | 7 (6.5) | 1 (5.6) | 2 (2.4) |  | 9 (5.8) | 1 (1.9) |  | 1 (2.6) | 5 (7.9) | 1 (2.4) | 3 (4.6) |  |
| Blank | 1 (0.5) | 0 | 1 (1.2) | 0 |  | 0 | 0 | 1 (1.2) |  | 0 | 1 (1.9) |  | 0 | 0 | 0 | 1 (1.5) |  |
| K8. Antibiotics can be used to cure infections caused by bacteria (Ans -True)^a^ | | | | | | | | | | | | | | | | | |
| True | 205 (98.6) | 107 (98.1) | 85 (98.8) | 13 (100) | 0.45 | 107 (99.1) | 18 (100) | 80 (97.6) | 0.79 | 153 (98.7) | 52 (98.1) | 0.30 | 38 (97.4) | 63 (100) | 40 (97.6) | 64 (98.5) | 0.38 |
| False | 2 (0.96) | 2 (1.8) | 0 | 0 |  | 1 (0.9) | 0 | 1 (1.2) |  | 2 (1.3) | 0 |  | 1 (2.6) | 0 | 1 (2.4) | 0 |  |
| Blank | 1 (0.5) | 0 | 1 (1.2) | 0 |  | 0 | 0 | 1 (1.2) |  | 0 | 1 (0.5) |  | 0 | 0 | 0 | 1 (1.54) |  |
| K9. Antibiotics can be used to cure infections caused by virus (Ans-False) | | | | | | | | | | | | | | | | | |
| True | 15 (7.2) | 9 (8.3) | 6 (7) | 0 | 0.92 | 8 (7.4) | 2 (11.1) | 5 (6.1) | 0.79 | 12 (7.7) | 3 (5.7) | 0.9 | 4 (10.3) | 4 (6.4) | 1 (2.4) | 6 (9.2) | 0.71 |
| False | 190 (91.4) | 98 (89.9) | 79 (91.9) | 13 (100) |  | 99 (91.7) | 16 (88.9) | 75 (91.5) |  | 141 (91) | 49 (92.5) |  | 34 (87.2) | 58 (92.1) | 40 (97.6) | 58 (89.2) |  |
| Blank | 3 (1.44) | 0 | 3 (1.2) | 0 |  | 1 (0.9) | 0 | 2 (2.4) |  | 2 (1.3) | 1 (1.9) |  | 1 (2.6) | 1 (1.6) | 0 | 1 (1.5) |  |
| K10. Do you think the use of antibiotics will speed up recovery of cold, cough and other diseases caused by common flu virus? (Ans-No)^b^ | | | | | | | | | | | | | | | | | |
| Yes | 70 (33.6) | 42 (38.5) | 25 (29.1) | 3 (23.1) | 0.29 | 38 (35.2) | 07 (38.9) | 25 (30.5) | 0.68 | 53 (34.2) | 17 (32.1) | 0.87 | 8 (20.5) | 30 (47.6) | 11 (26.8) | 21 (32.3) | 0.03 |
| No | 138 (66.4) | 67 (61.5) | 61 (70.9) | 10 (76.9) |  | 70 (64.8) | 11 (61.1) | 57 (69.5) |  | 102 (65.8) | 36 (67.9) |  | 31 (79.5) | 33 (52.4) | 30 (73.2) | 44 (67.7) |  |
| K11. Do you think frequent prescribe of antibiotics will decrease the efficacy of drug? (Ans-Yes)^a^ | | | | | | | | | | | | | | | | | |
| Yes | 203 (97.6) | 107 (98.2) | 83 (96.5) | 13 (100) | 0.51 | 105 (97.2) | 18 (100) | 80 (97.6) | 1.0 | 152 (98.1) | 51 (96.2) | 0.29 | 38 (97.4) | 61 (96.8) | 41 (100) | 63 (96.9) | 0.46 |
| No | 4 (1.9) | 1 (0.9) | 3 (3.5) | 0 |  | 2 (1.9) | 0 | 2 (2.5) |  | 3 (1.9) | 1 (1.9) |  | 0 | 2 (3.2) | 0 | 2 (3.1) |  |
| Blank | 1 (0.5) | 1 (0.9) | 0 | 0 |  | 1 (0.9) | 0 | 0 |  | 0 | 1 (1.9) |  | 1 (2.6) | 0 | 0 | 0 |  |
| K12. Do you think antibiotic should be used for disease prevention? (Ans-No)^b^ | | | | | | | | | | | | | | | | | |
| Yes | 18 (8.7) | 8 (7.3) | 7 (8.1) | 3 (23.1) | 0.17 | 8 (7.4) | 1 (5.6) | 9 (10.9) | 0.65 | 14 (9) | 4 (7.6) | 1.00 | 4 (10.3) | 5 (7.9) | 1 (2.4) | 8 (12.3) | 0.32 |
| No | 190 (91.4) | 101 (92.7) | 79 (91.9) | 10 (76.4) |  | 100 (92.6) | 17 (94.4) | 73 (89) |  | 141 (91) | 49 (92.5) |  | 35 (89.7) | 58 (92.1) | 40 (97.6) | 57 (87.7) |  |
| K13. Do you think antibiotic drug residues and drug resistance will occur when antibiotics are not used prudently?^a^ | | | | | | | | | | | | | | | | | |
| Yes | 104 (95.4) | 101 (92.7) | 75 (87.2) | 9 (69.2) | 0.003 | 94 (87.04) | 17 (94.4) | 77 (93.9) | 0.19 | 140 (90.3) | 48 (90.6) | 0.33 | 37 (94.9) | 58 (92.1) | 38 (92.7) | 55 (84.6) | 0.57 |
| No | 5 (4.6) | 8 (7.3) | 11 (12.8) | 3 (23.1) |  | 14 (12.96) | 1 (5.6) | 4 (4.9) |  | 15 (9.7) | 4 (7.5) |  | 2 (5.1) | 5 (7.9) | 3 (7.3) | 9 (13.9) |  |
| Blank | 0 (0) | 0 (0) | 0 (0) | 1 (7.7) |  | 0 (0) | 0 (0) | 1 (1.2) |  | 0 (0) | 1 (1.9) |  | 0 (0) | 0 (0) | 0 (0) | 1 (1.5) |  |
| K14. Do you think biosecurity and improved hygiene can reduce the use of antibiotics? (Ans-Yes) | | | | | | | | | | | | | | | | | |
| Yes | 208 (100) | 109 (100) | 86 (100) | 13 (100) |  | 108 (100) | 18 (100) | 82 (100) |  | 155 (100) | 53 (100) |  | 39 (100) | 63 (100) | 41 (100) | 65 (100) |  |
| No | 0 | 0 | 0 | 0 |  | 0 | 0 | 0 |  | 0 | 0 |  | 0 | 0 | 0 | 0 |  |

^a^Fisher’s Exact Test

^b^Chi-square Test
