# Supplementary material for: Knowledge, attitude and practice towards antibiotic use and resistance among the veterinarians in Bangladesh
Source: PLoS One. 2024 Aug 13;19(8):e0308324. doi: 10.1371/journal.pone.0308324 (PMC11321546; doi:10.1371/journal.pone.0308324)
Supplement: S2 Table — (DOCX) [file pone.0308324.s003.docx]

**Table S3. Veterinarian’s attitude towards antibiotic use and resistance**

| **Attitude statements** | **Overall n (%)** | **Age groups (Year)**  **n (%)** | | | | **Field of expertise**  **n (%)** | | | | **Type of service**  **n (%)** | | | **Years of practice**  **n (%)** | | | | |
| --- | --- | --- | --- | --- | --- | --- | --- | --- | --- | --- | --- | --- | --- | --- | --- | --- | --- |
|  |  | **25-30** | **31-35** | **>36** | **P value** | **Poultry** | **Pet animals** | **Large & small  animals'** | **P value** | **Private** | **Government** | **P value** | **<1** | **1-3** | **>3-5** | **>5** | **P value** |
| A1. In your opinion, only veterinarians are eligible for drugs prescriptions for animals?^a^ | | | | | | | | | | | | | | | | | |
| Yes | 207 (99.5) | 108 (99.1) | 86 (100) | 13 (100) | 1.0 | 108 (100) | 18 (100) | 81 (98.8) | 0.48 | 154 (99.4) | 53 (100) | 1.0 | 39 (100) | 62 (98.4) | 41 (100) | 65 (100) | 0.69 |
| No | 1 (0.5) | 1 (0.9) | 0 | 0 |  | 0 | 0 | 1 (1.2) |  | 1 (0.7) | 0 |  | 0 | 1 (1.6) | 0 | 0 |  |
| A2. Use of antibiotic^a^ | | | | | | | | | | | | | | | | | |
| For therapeutic reasons | 201 (96.6) | 106 (97.3) | 82 (95.4) | 13 (100) | 0.81 | 105 (97.2) | 18 (100) | 78 (95.1) | 0.72 | 150 (96.8) | 51 (96.2) | 1.0 | 38 (97.4) | 61 (96.8) | 40 (97.6) | 62 (95.4) | 1.0 |
| For prophylactic reasons | 7 (3.4) | 3 (2.8) | 4 (4.7) | 0 |  | 3 (2.8) | 0 | 4 (4.9) |  | 5 (3.2) | 2 (3.8) |  | 1 (2.6) | 2 (3.2) | 1 (2.4) | 3 (4.6) |  |
| To increase growth rates or production | 0 | 0 | 0 | 0 |  | 0 | 0 | 0 |  | 0 | 0 |  | 0 | 0 | 0 | 0 |  |
| A3. At present, there is abuse of antibiotics^a^ | | | | | | | | | | | | | | | | | |
| Yes | 205 (98.6) | 107 (98.2) | 85 (98.8) | 13 (100) | 1.0 | 106 (98.2) | 18 (100) | 81 (98.8) | 1.0 | 152 (98.1) | 53 (100) | 0.57 | 38 (97.4) | 63 (100) | 40 (97.6) | 64 (98.5) | 0.60 |
| No | 3 (1.4) | 2 (1.8) | 1 (1.2) | 0 |  | 2 (1.9) | 0 | 1 (1.2) |  | 3 (1.9) | 0 |  | 1 (2.6) | 0 | 1 (2.4) | 1 (1.5) |  |
| A4. Antibiotic resistance affects you and your family’s health^a^ | | | | | | | | | | | | | | | | | |
| Agree | 206 (99) | 108 (99.1) | 85 (98.8) | 13 (100) | 1.0 | 106 (98.2) | 18 (100) | 82 (100) | 0.59 | 153 (98.7) | 53 (100) | 1.0 | 39 (100) | 62 (98.4) | 41 (100) | 64 (98.5) | 1.0 |
| Disagree | 0 | 0 | 0 | 0 |  | 0 | 0 | 0 |  | 0 | 0 |  | 0 | 0 | 0 | 0 |  |
| Neutral | 2 (1) | 1 (0.9) | 1 (1.2) | 0 |  | 2 (1.9) | 0 | 0 |  | 2 (1.3) | 0 |  | 0 | 1 (1.6) | 0 | 1 (1.5) |  |
| A5. When a disease in individual can't be treated with antibiotics, how serious do you think it could be?^a^ | | | | | | | | | | | | | | | | | |
| Very serious | 119 (57.2) | 67 (61.5) | 46 (53.5) | 6 (46.2) | 0.09 | 52 (48.2) | 13 (72.2) | 54 (65.9) | 0.05 | 82 (52.9) | 37 (69.8) | 0.006 | 23 (59) | 35 (55.6) | 27 (65.9) | 34 (52.3) | 0.02 |
| Serious | 53 (25.5) | 24 (22) | 26 (30.2) | 3 (23.1) |  | 29 (26.9) | 5 (27.8) | 19 (23.2) |  | 43 (27.7) | 10 (18.9) |  | 6 (15.4) | 22 (34.9) | 11 (26.8) | 14 (21.5) |  |
| Less serious | 11 (5.3) | 9 (8.3) | 2 (2.3) | 0 |  | 9 (8.3) | 0 | 2 (2.4) |  | 7 (4.5) | 4 (7.6) |  | 5 (12.8) | 3 (4.8) | 0 | 3 (4.6) |  |
| Not serious at all | 24 (11.5) | 8 (7.3) | 12 (14) | 4 (30.8) |  | 18 (16.7) | 0 | 6 (7.3) |  | 23 (14.8) | 1 (1.9) |  | 4 (10.3) | 3 (4.8) | 0 | 14 (21.5) |  |
| Blank | 1 (0.5) | 1 (0.9) | 0 | 0 |  | 0 | 0 | 1 (1.2) |  | 0 | 1 (1.9) |  | 1 (2.6) | 0 | 0 | 0 |  |
| A6. When a disease in animal cannot be treated with antibiotics, how serious do you think it could be?^a^ | | | | | | | | | | | | | | | | | |
| Very serious | 114 (54.8) | 64 (58.7) | 44 (51.2) | 6 (46.2) | 0.20 | 52 (48.2) | 12 (6.7) | 50 (61) | 0.48 | 81 (52.3) | 33 (62.3) | 0.27 | 24 (61.5) | 34 (54) | 21 (51.2) | 35 (53.9) | 0.03 |
| Serious | 60 (28.9) | 28 (25.7) | 29 (33.7) | 3 (23.1) |  | 33 (30.6) | 6 (33.3) | 21 (25.6) |  | 45 (29) | 15 (28.3) |  | 5 (12.8) | 23 (36.5) | 17 (41.5) | 15 (23.1) |  |
| Less serious | 10 (4.8) | 8 (7.3) | 2 (2.3) | 0 |  | 7 (6.5) | 0 | 3 (3.7) |  | 8 (5.2) | 2 (3.8) |  | 4 (10.3) | 4 (6.4) | 0 | 2 (3.1) |  |
| Not serious at all | 22 (10.6) | 8 (7.3) | 10 (11.6) | 4 (30.8) |  | 15 (13.9) | 0 | 7 (8.5) |  | 20 (12.9) | 2 (3.8) |  | 5 (12.8) | 2 (3.2) | 3 (7.3) | 12 (18.5) |  |
| Blank | 2 (0.96) | 1 (0.9) | 1 (1.2) | 0 |  | 1 (0.9) | 0 | 1 (1.2) |  | 1 (0.7) | 1 (1.9) |  | 1 (2.6) | 0 | 0 | 1 (1.5) |  |
| A7. Do you think vaccination can prevent disease?^a^ | | | | | | | | | | | | | | | | | |
| Yes | 207 (99.5) | 108 (99.1) | 86 (100) | 13 (100) | 1.00 | 107 (99.1) | 18 (100) | 82 (100) | 1.0 | 154 (99.4) | 53 (100) | 1.0 | 39 (100) | 63 (100) | 40 (97.6) | 65 (100) | 0.39 |
| No | 1 (0.5) | 1 (0.9) | 0 | 0 |  | 1 (0.9) | 0 | 0 |  | 1 (0.7) | 0 |  | 0 | 0 | 1 (2.4) | 0 |  |
| A8. Do you think vaccination can reduce the uses of antibiotics?^a^ | | | | | | | | | | | | | | | | | |
| Yes | 203 (97.6) | 105 (96.3) | 85 (98.8) | 13 (100) | 0.56 | 104 (96.3) | 18 (100) | 81 (98.8) | 0.62 | 150 (96.8) | 53 (100) | 0.33 | 37 (94.9) | 62 (98.4) | 40 (97.6) | 64 (98.5) | 0.72 |
| No | 5 (2.4) | 4 (3.7) | 1 (1.2) | 0 |  | 4 (3.7) | 0 | 1 (1.2) |  | 5 (3.2) | 0 |  | 2 (5.1) | 1 (1.6) | 1 (2.4) | 1 (1.5) |  |
| A9. Is it necessary to establish a law on “Rational use of antibiotics” at the national level?^a^ | | | | | | | | | | | | | | | | | |
| Yes | 206 (99) | 107 (98.2) | 86 (100) | 13 (100) | 0.57 | 106 (98.2) | 18 (100) | 82 (100) | 0.59 | 153 (98.7) | 53 (100) | 1.0 | 39 (100) | 62 (98.4) | 41 (100) | 64 (98.5) | 1.0 |
| No | 2 (1) | 2 (1.8) | 0 | 0 |  | 2 (1.9) | 0 | 0 |  | 2 (1.3) | 0 |  | 0 | 1 (1.6) | 0 | 1 (1.5) |  |
| A10. A local antimicrobial guideline would be more useful than international one^a^ | | | | | | | | | | | | | | | | | |
| Yes | 181 (87) | 91 (83.5) | 79 (91.9) | 11 (84.6) | 0.3 | 91 (84.3) | 17 (94.4) | 73 (89) | 0.38 | 131 (84.5) | 50 (94.3) | 0.16 | 33 (84.6) | 55 (87.3) | 35 (85.4) | 58 (89.2) | 0.79 |
| No | 26 (12.5) | 17 (15.6) | 7 (8.1) | 2 (15.4) |  | 17 (15.7) | 1 (5.6) | 8 (9.8) |  | 23 (14.8) | 3 (5.7) |  | 5 (12.8) | 8 (12.7) | 6 (14.6) | 7 (10.8) |  |
| Blank | 1 (0.5) | 1 (0.9) | 0 | 0 |  | 0 | 0 | 1 (1.2) |  | 1 (0.7) | 0 |  | 1 (2.6) | 0 | 0 | 0 |  |
| A11. It is important to add antibiotic with feed/water as growth promoter in livestock^a^ | | | | | | | | | | | | | | | | | |
| Agree | 10 (4.8) | 2 (1.8) | 8 (9.3) | 0 | 0.11 | 3 (2.8) | 1 (5.6) | 6 (7.3) | 0.44 | 8 (5.2) | 2 (3.8) | 0.51 | 1 (2.6) | 3 (4.8) | 0 | 6 (9.2) | 0.25 |
| Disagree | 165 (79.3) | 89 (81.7) | 67 (77.9) | 9 (69.2) |  | 90 (83.3) | 15 (83.3) | 60 (73.2) |  | 119 (76.8) | 46 (86.8) |  | 28 (71.8) | 50 (79.4) | 36 (87.8) | 51 (78.5) |  |
| Neutral | 32 (15.4) | 17 (15.6) | 11 (12.8) | 4 (30.8) |  | 15 (13.9) | 2 (11.1) | 15 (18.3) |  | 27 (17.4) | 5 (9.4) |  | 9 (23.1) | 10 (15.9) | 5 (12.2) | 8 (12.3) |  |
| Blank | 1 (0.5) | 1 (0.9) | 0 | 0 |  | 0 | 0 | 1 (1.2) |  | 1 (0.7) | 0 |  | 1 (2.6) | 0 | 0 | 0 |  |
| A12. Inappropriate use or half course of antibiotics leads to antibiotic resistance^a^ | | | | | | | | | | | | | | | | | |
| Agree | 203 (97.6) | 108 (99.1) | 85 (98.8) | 10 (76.9) | 0.001 | 106 (98.2) | 18 (100) | 79 (96.3) | 0.72 | 151 (97.4) | 52 (98.1) | 0.29 | 38 (97.4) | 62 (98.4) | 41 (100) | 62 (95.4) | 0.63 |
| Disagree | 1 (0.5) | 0 | 0 | 1 (7.7) |  | 0 | 0 | 1 (1.2) |  | 0 | 1 (1.9) |  | 0 | 0 | 0 | 1 (1.5) |  |
| Neutral | 4 (1.9) | 1 (0.9) | 1 (1.2) | 2 (15.4) |  | 2 (1.9) | 0 | 2 (2.4) |  | 4 (2.6) | 0 |  | 1 (2.6) | 1 (1.6) | 0 | 2 (3.1) |  |
| Blank | 0 | 0 | 0 | 0 |  | 0 | 0 | 0 |  | 0 | 0 |  | 0 | 0 | 0 | 0 |  |
| A13. An appropriate withdrawal period is needed before selling to avoid antibiotic residue in food animal?^a^ | | | | | | | | | | | | | | | | | |
| Agree | 205 (98.6) | 108 (99.1) | 84 (97.7) | 13 (100) | 0.34 | 107 (99.1) | 18 (100) | 80 (97.6) | 0.79 | 152 (98.1) | 53 (100) | 1.0 | 38 (97.4) | 63 (100) | 40 (97.6) | 64 (98.5) | 0.38 |
| Disagree | 2 (1) | 0 | 2 (2.3) | 0 |  | 1 (0.9) | 0 | 1 (1.2) |  | 2 (1.3) | 0 |  | 0 | 0 | 1 (2.4) | 1 (1.5) |  |
| Neutral | 1 (0.5) | 1 (0.9) | 0 | 0 |  | 0 | 0 | 1 (1.2) |  | 1 (0.7) | 0 |  | 1 (2.6) | 0 | 0 | 0 |  |
| Blank | 0 | 0 | 0 | 0 |  |  |  |  |  | 0 | 0 |  | 0 | 0 | 0 | 0 |  |
| A15. Have you ever attended any training/conference/seminar/workshop on antimicrobial resistance?^b^ | | | | | | | | | | | | | | | | | |
| Yes | 127 (61.1) | 69 (63.3) | 49 (57) | 9 (69.2) | 0.549 | 56 (51.9) | 11 (61.1) | 60 (73.2) | 0.01 | 86 (55.5) | 41 (77.4) | 0.01 | 32 (82.1) | 31 (49.2) | 26 (63.4) | 38 (58.5) | 0.01 |
| No | 81 (38.9) | 40 (36.7) | 37 (43.) | 4 (30.8) |  | 52 (48.2) | 7 (38.9) | 22 (26.8) |  | 69 (44.5) | 12 (22.6) |  | 7 (18) | 32 (50.8) | 15 (36.6) | 27 (41.5) |  |

^a^Fisher’s Exact Test

^b^Chi-square Test
