# Supplementary material for: Knowledge, attitude and practice towards antibiotic use and resistance among the veterinarians in Bangladesh
Source: PLoS One. 2024 Aug 13;19(8):e0308324. doi: 10.1371/journal.pone.0308324 (PMC11321546; doi:10.1371/journal.pone.0308324)
Supplement: S3 Table — (DOCX) [file pone.0308324.s004.docx]

**Table S4. Veterinarian’s practice regarding antibiotic use and resistance**

| **Practice statements** | **Overall**  **n (%)** | **Age groups (Year)**  **n (%)** | | | | **Field of expertise**  **n (%)** | | | | **Type of service**  **n (%)** | | | **Years of practice**  **n (%)** | | | | |
| --- | --- | --- | --- | --- | --- | --- | --- | --- | --- | --- | --- | --- | --- | --- | --- | --- | --- |
|  |  | **25-30** | **31-35** | **>36** | **P value** | **Poultry** | **Pet animals** | **Large & small  animals** | **P value** | **Private** | **Government** | **P value** | **0-1** | **1-3** | **3-5** | **>5** | **P value** |
| P1. Do you prescribe antibiotics over phone or without seeing/examining animals?^a^ | | | | | | | | | | | | | | | | | |
| Often/Always | 6 (2.9) | 5 (4.6) | 1 (1.2) | 0 | 0.25 | 2 (1.85) | 0 | 4 (4.8) | 0.82 | 5 (3.2) | 1 (1.9) | 1.0 | 2 (5.1) | 3 (4.7) | 0 | 1 (1.5) | 0.67 |
| Sometimes | 146 (70.2) | 70 (64.2) | 67 (77.9) | 9 (69.2) |  | 76 (70.4) | 13 (72.2) | 57 (69.5) |  | 108 (69.7) | 38 (71.7) |  | 25 (64.1) | 42 (66.7) | 30 (73.2) | 49 (75.4) |  |
| Never/rarely | 56 (26.9) | 34 (31.2) | 18 (20.9) | 4 (30.8) |  | 30 (27.8) | 5 (27.8) | 21 (25.6) |  | 42 (27.1) | 14 (26.4) |  | 12 (30.8) | 18 (28.6) | 11 (26.8) | 15 (23.1) |  |
| P2. Do you have facilities in your area to test antimicrobial sensitivity?^b^ | | | | | | | | | | | | | | | | | |
| Yes | 93 (44.7) | 46 (42.2) | 43 (50) | 4 (30.8) | 0.32 | 49 (45.4) | 12 (66.7) | 32 (39) | 0.1 | 76 (49) | 17 (32.1) | 0.03 | 12 (30.8) | 36 (57.14) | 14 (34.2) | 31 (47.7) | 0.03 |
| No | 115 (55.3) | 63 (57.8) | 43 (50) | 9 (69.2) |  | 59 (54.6) | 6 (33.3) | 50 (61) |  | 79 (51) | 36 (68) |  | 27 (69.2) | 27 (42.9) | 27 (65.9) | 34 (52.3) |  |
| P3. How often on a first visit to a farm you suggest antimicrobial susceptibility testing if you suspect a bacterial infection is present?^b^ | | | | | | | | | | | | | | | | | |
| Often/Always | 19 (9.1) | 8 (7.3) | 10 (11.6) | 1 (7.69) | 0.85 | 9 (8.3) | 1 (05.6) | 09 (11) | 0.04 | 15 (9.7) | 4 (7.6) | 0.37 | 4 (10.3) | 5 (8) | 4 (9.7) | 6 (9.2) | 0.87 |
| Sometimes | 104 (50) | 57 (52.29) | 41 (47.7) | 6 (46.2) |  | 60 (55.6) | 13 (72.2) | 31 (37.8) |  | 81 (52.3) | 23 (43.4) |  | 16 (40) | 35 (55.6) | 22 (53.7) | 31 (47.7) |  |
| Never/rarely | 85 (40.9) | 44 (40.4) | 35 (40.7) | 06 (46.2) |  | 39 (36.1) | 04 (22.2) | 42 (51.2) |  | 59 (38.1) | 26 (49.1) |  | 19 (48.7) | 23 (36.5) | 15 (36.6) | 28 (43.1) |  |
| P4. How often do you suggest antimicrobial susceptibility testing, when a pathogen has not responded to the first antibiotic used?^b^ | | | | | | | | | | | | | | | | | |
| Often/Always | 41 (19.7) | 25 (22.9) | 14 (16.3) | 2 (15.4) | 0.49 | 29 (26.9) | 2 (11.1) | 10 (12.2) | 0.03 | 34 (21.9) | 7 13.2) | 0.38 | 11 (28.2) | 11 (17.5) | 8 (19.5) | 11 (16.9) | 0.66 |
| Sometimes | 111 (53.4) | 60 (55.1) | 44 (51.2) | 7 (53.9) |  | 55 (50.9) | 13 (72.2) | 43 (52.4) |  | 81 (52.3) | 30 (56.6) |  | 21 (53.9) | 36 (57.1) | 21 (51.2) | 33 (50.8) |  |
| Never/rarely | 56 (26.9) | 24 (22) | 28 (32.6) | 4 (30.8) |  | 24 (22.2) | 3 (16.7) | 29 (35.4) |  | 40 (25.8) | 16 (30.2) |  | 7 (18) | 16 (24.4) | 12 (29.3) | 21 (32.3) |  |
| P5. How often do you encounter a poor clinical response to an antimicrobial used?^a^ | | | | | | | | | | | | | | | | | |
| Often/Always | 19 (9.1) | 14 (12.8) | 5 (5.8) | 0 | 0.24 | 10 (9.3) | 0 | 9 (11) | 0.67 | 2 (1.3) | 0 | 0.95 | 1 (02.6) | 1 (1.6) | 0 | 0 | 0.6 |
| Sometimes | 170 (81.7) | 82 (75.2) | 75 (87.2) | 13 (100) |  | 90 (83.3) | 17 (94.4) | 63 (76.8) |  | 14 (9) | 5 (9.4) |  | 5 (12.8) | 6 (9.5) | 4 (9.8) | 4 (6.2) |  |
| Never/rarely | 17 (8.2) | 11 (10.1) | 6 (7) | 0 |  | 7 (6.5) | 1 (5.6) | 9 (11) |  | 127 (81.9) | 43 (81.1) |  | 28 (71.8) | 51 (81) | 33 (80.5) | 58 (89.2) |  |
| Blank | 2 (1) | 2 (1.8) | 0 | 0 |  | 1 (0.9) | 0 | 1 (1.2) |  | 12 (7.7) | 5 (9.4) |  | 5 (12.8) | 5 (07.9) | 4 (9.8) | 3 (4.6) |  |
| P6. How commonly do you feel that poor clinical response may be due to antimicrobial resistance?^a^ | | | | | | | | | | | | | | | | | |
| Often/Always | 76 (36.5) | 48 (44) | 24 (27.9) | 4 (30.8) | 0.05 | 48 (44.4) | 2 (11.1) | 26 (31.7) | 0.01 | 58 (37.4) | 18 (34) | 0.67 | 0 | 1 (1.6) | 0 | 0 | 0.11 |
| Sometimes | 124 (59.6) | 56 (51.4) | 60 (69.8) | 8 (61.5) |  | 59 (54.6) | 15 (83.3) | 50 (61) |  | 92 (59.4) | 32 (60.4) |  | 19 (48.7) | 23 (36.1) | 18 (43.9) | 16 (24.6) |  |
| Never/rarely | 7 (3.4) | 5 (4.6) | 1 (1.2) | 1 (7.7) |  | 1 (0.9) | 1 (5.6) | 5 (6.1) |  | 4 (2.6) | 3 (5.7) |  | 19 (48.7) | 36 (57.1) | 21 (51.2) | 48 (73.9) |  |
| Blank | 1 (0.5) | 0 | 1 (1.2) | 0 |  | 0 | 0 | 1 (1.2) |  | 1 (0.7) | 0 |  | 1 (2.6) | 3 (4.8) | 2 (04.9) | 1 (01.5) |  |
| P7. How often have you had to change an antimicrobial agent because of resistance confirmed on antimicrobial susceptibility testing?^a^ | | | | | | | | | | | | | | | | | |
| Often/Always | 50 (24) | 23 (21.1) | 23 (26.7) | 4 (30.8) | 0.81 | 33 (30.6) | 2 (11.1) | 15 (18.3) | 0.16 | 38 (24.5) | 12 (22.6) | 0.39 | 12 (30.8) | 10 (15.8) | 8 (19.5) | 20 (30.8) | 0.47 |
| Sometimes | 118 (56.7) | 65 (59.6) | 46 (53.5) | 7 (53.9) |  | 56 (51.9) | 14 (77.8) | 48 (58.5) |  | 91 (58.7) | 27 (50.9) |  | 21 (53.9) | 39 (61.9) | 26 (63.4) | 32 (49.2) |  |
| Never/rarely | 39 (18.8) | 21 (19.3) | 16 (18.6) | 2 (15.4) |  | 18 (16.7) | 2 (11.1) | 19 (23.2) |  | 25 (16.1) | 14 (26.4) |  | 6 (15.4) | 14 (22.2) | 7 (17) | 12 (18.5) |  |
| Blank | 1 (0.5) | 0 | 1 (1.2) | 0 |  | 1 (0.3) | 0 | 0 |  | 01 (0.6) | 0 |  | 0 | 0 | 0 | 1 01.5) |  |
| P9. Which spectrum of antibiotics do you prefer most?^b^ | | | | | | | | | | | | | | | | | |
| Broad spectrum | 104 (50) | 55 (50.5) | 41 (47.7) | 8 (61.5) | 0.64 | 60 (55.6) | 6 (33.3) | 38 (46.3) | 0.15 | 81 (52.3) | 23 (43.4) | 0.27 | 22 (56.1) | 29 (46) | 16 (39) | 37 (56.9) | 0.24 |
| Narrow spectrum | 104 (50) | 54 (49.5) | 45 (52.3) | 5 (38.5) |  | 48 (44.4) | 12 (66.7) | 44 (53.7) |  | 74 (47.7) | 30 (56.6) |  | 17 (43.6) | 34 (54) | 25 (61) | 28 (43.1) |  |
| P10. What percentage of your daily prescriptions has antibiotics?^a^ | | | | | | | | | | | | | | | | | |
| <20 | 54 (26) | 29 (26.6) | 21 (24.42) | 4 (30.8) | 0.97 | 27 (25) | 8 (44.4) | 19 (23.2) | 0.28 | 47 (30.3) | 7 (13.2) | 0.04 | 10 (25.6) | 19 (30.16) | 12 (29.27) | 13 (20) | 0.43 |
| 20% to 40% | 95 (45.7) | 48 (44) | 41 (47.7) | 6 (46.2) |  | 47 (43.5) | 8 (44.4) | 40 (48.8) |  | 66 (42.6) | 29 (54.7) |  | 15 (38.5) | 32 (50.79) | 18 (43.90) | 30 (46.2) |  |
| 41% to 60% | 59 (28.4) | 32 (29.4) | 24 (27.9) | 3 (23.1) |  | 34 (31.5) | 2 (11.1) | 23 (28.1) |  | 42 (27.1) | 17 (32.1) |  | 14 (35.9) | 12 (19.1) | 11 (26.8) | 22 (33.9) |  |
| Above 60% | 0 | 0 | 0 | 0 |  | 0 | 0 | 0 |  | 0 | 0 |  |  |  |  |  |  |
| P11. Do you mention withdrawal period in the prescription?^b^ | | | | | | | | | | | | | | | | | |
| Yes | 53 (25.5) | 35 (32.1) | 16 (18.6) | 2 (15.4) | 0.07 | 20 (18.5) | 08 (44.4) | 25 (30.5) | 0.03 | 40 (25.8) | 13 (24.5) | 0.85 | 15 (38.5) | 19 (30.2) | 8 (19.5) | 11 (16.9) | 0.06 |
| No | 155 (74.5) | 74 (67.9) | 70 (81.4) | 11 (84.6) |  | 88 (81.5) | 10 (55.6) | 57 (69.5) |  | 115 (74.2) | 40 (75.5) |  | 24 (61.5) | 44 (69.8) | 33 (80.5) | 54 (83.1) |  |
| P12. Do you suggest keeping drug register of animals?^a^ | | | | | | | | | | | | | | | | | |
| Yes | 155 (74.5) | 83 (76.2) | 65 (75.6) | 7 (53.9) | 0.07 | 83 (76.9) | 15 (83.3) | 57 (69.5) | 0.44 | 114 (73.6) | 41 (77.4) | 0.79 | 27 (69.2) | 45 (71.4) | 34 (82.9) | 49 (75.4) | 0.54 |
| No | 52 (25) | 26 (23.9) | 21 (24.4) | 5 (38.5) |  | 24 (22.2) | 03 (16.7) | 25 (30.5) |  | 40 (25.8) | 12 (22.6) |  | 12 (30.8) | 18 (28.6) | 7 (17.1) | 15 (23.1) |  |
| Blank | 1(0.5) | 0 | 0 | 1 (7.69) |  | 1 (0.9) | 0 | 0 |  | 1 (0.7) | 0 |  | 0 | 0 | 0 | 1 (1.5) |  |
| P13. Do you prefer combine antibiotics to ensure therapeutic success?^b^ | | | | | | | | | | | | | | | | | |
| Yes | 135 (64.9) | 70 (64.2) | 57 (66.3) | 8 (61.5) | 0.92 | 67 (62) | 8 (44.4) | 60 (73.2) | 0.05 | 102 (65.8) | 33 (62.3) | 0.64 | 28 (71.8) | 40 (63.5) | 23 (56.1) | 44 (67.7) | 0.48 |
| No | 73 (35.1) | 39 (35.8) | 29 (33.7) | 5 (38.5) |  | 41 (38) | 10 (55.6) | 22 (26.8) |  | 53 (34.2) | 20 (37.7) |  | 11 (28.2) | 23 (36.5) | 18 (43.9) | 21 (32.3) |  |
| P14. Do you administer antibiotics to animals without determining their body weight properly?^a^ | | | | | | | | | | | | | | | | | |
| Yes | 54 (26) | 28 (25.7) | 22 (25.6) | 4 (30.8) | 0.95 | 23 (21.3) | 04 (22.2) | 27 (32.9) | 0.3 | 44 (28.4) | 10 (18.8) | 0.41 | 11 (28.2) | 16 (25.4) | 12 (29.3) | 15 (23.1) | 0.93 |
| No | 153 (73.6) | 80 (73.4) | 64 (74.4) | 9 (69.2) |  | 84 (77.8) | 14 (77.8) | 55 (67.1) |  | 110 (7) | 43 (81.1) |  | 28 (71.8) | 46 (73.1) | 29 (70.7) | 50 (76.9) |  |
| Blank | 1 (0.5) | 1 (0.9) | 0 | 0 |  | 1 (00.9) | 0 | 0 |  | 1 (0.7) | 0 |  | 0 | 1 (1.6) | 0 | 0 |  |
| P15. Do you consider whether an infection is self-limiting before prescribing antibiotics?^a^ | | | | | | | | | | | | | | | | | |
| Yes | 162 (77.9) | 93 (85.3) | 59 (68.6) | 10 (76.9) | 0.03 | 82 (75.9) | 16 (88.9) | 64 (78.1) | 0.78 | 116 (74.8) | 46 (86.8) | 0.07 | 33 (84.6) | 47 (74.6) | 32 (78.1) | 50 (76.9) | 0.64 |
| No | 46 (22.1) | 16 (14.7) | 25 (29.1) | 3 (23.1) |  | 26 (24.1) | 2 (11.1) | 18 (22) |  | 39 (25.2) | 7 (13.2) |  | 6 (15.4) | 16 (25.4) | 9 (22) | 15 (23.1) |  |
| Blank | 0 | 0 | 2 (2.3) | 0 |  | 0 | 0 | 0 |  | 0 | 0 |  | 0 | 0 | 0 | 0 |  |
| P16. As the first line of treatment, do you choose new generation antibiotics rather than older generations like penicillin?^b^ | | | | | | | | | | | | | | | | | |
| Yes | 76 (36.5) | 34 (31.2) | 36 (41.9) | 6 (46.2) | 0.23 | 45 (41.7) | 5 (27.8) | 26 (31.7) | 0.27 | 62 (40) | 14 (26.4) | 0.08 | 13 (33.3) | 19 (30.2) | 12 (29.3) | 32 (49.2) | 0.08 |
| No | 132 (63.5) | 75 (68.8) | 50 (58.1) | 7 (53.9) |  | 63 (58.3) | 13 (72.2) | 56 (68.3) |  | 93 (60) | 39 (73.6) |  | 26 (66.7) | 44 (69.8) | 29 (70.7) | 33 (50.8) |  |
| P17. Do you consult with other veterinarian/other educational resources when in doubt of a drug’s mechanism of action?^a^ | | | | | | | | | | | | | | | | | |
| Yes | 199 (95.7) | 104 (94.4) | 83 (96.5) | 12 (92.3) | 0.62 | 104 (96.3) | 17 (94.4) | 78 (95.1) | 0.68 | 151 (97.4) | 48 (90.6) | 0.05 | 37 (94.8) | 61 (96.8) | 41 (100) | 60 (92.3) | 0.24 |
| No | 9 (4.3) | 5 (4.6) | 3 (3.5) | 1 (7.7) |  | 4 (3.7) | 1 (5.6) | 4 (4.9) |  | 4 (2.6) | 5 (9.4) |  | 2 (5.1) | 2 (3.2) | 0 | 5 (7.7) |  |
| P18. Do you suggest clients to vaccinate their animals against preventable diseases?^a^ | | | | | | | | | | | | | | | | | |
| Yes | 205 (98.6) | 108 (99.1) | 86 (100) | 11 (84.6) | 0.01 | 106 (98.2) | 18 (100) | 81 (98.8) | 1.0 | 152 (98.1) | 53 (100) | 0.57 | 38 (97.4) | 63 (100) | 41 (100) | 63 (96.9) | 0.41 |
| No | 3 (1.44) | 1 (0.9) | 0.00 | 2 (15.4) |  | 2 (1.9) | 0 | 1 (1.2) |  | 03 (1.9) | 0 |  | 1 (2.56) | 0 | 0 | 2 (3.1) |  |

^a^Fisher’s Exact Test

^b^Chi-square Test
